# Traders, guns, and money: The effects of mass shootings on stock prices of firearm manufacturers in the U.S.

**DOI:** 10.1371/journal.pone.0177720

**Published:** 2017-05-18

**Authors:** Anandasivam Gopal, Brad N. Greenwood

**Affiliations:** 1 Robert H Smith School of Business, University of Maryland–College Park, College Park, MD, United States of America; 2 Fox School of Business, Temple University, Philadelphia, PA, United States of America; Iowa State University, UNITED STATES

## Abstract

We investigate how mass shootings influence the stock price of firearms manufacturers. While it is well known that mass shootings lead to increased firearms sales, the response from financial markets is unclear. On one hand, given the observed short-term increase in demand, firearm stock prices may rise due to the unexpected financial windfall for the firm. On the other, mass shootings may result in calls for regulation of the industry, leading to divestment of firearms stocks in spite of short-term demand. We examine this tension using a market movement event study in the wake of 93 mass shootings in the U.S. between 2009 and 2013. Findings show that stock prices of firearm manufacturers decline after shootings; each event reducing prices between 22.4 and 49.5 basis points, per day. These losses are exacerbated by the presence of a handgun and the number of victims killed, but not affected by the presence of children or location of the event. Finally, we find that these effects are most prevalent in the period 2009–2010 but disappear in later events, indicating that markets appear to have accepted mass shootings as the “new normal.”

## Introduction

In recent years, the American public has been continually traumatized by mass shootings; events where individuals suffering from mental health issues or psychological trauma and access to firearms open fire on strangers in seemingly senseless acts of violence [1]. Although scholars and policy makers have speculated at length regarding the impetus for such events, one plausible explanation which continually has surfaced is the easy access to firearms in the US [2]. In 2013, for example the *Washington Post* reported that among OECD countries, the US had the highest number of firearms per capita, as well as the highest gun-related deaths per capita [3]. However, while the origin of such events has been studied extensively (the mental health of shooters being indicted as the primary catalyst [4]), one aspect of mass shootings and gun violence remains understudied: the response of financial markets to such events. More specifically, if firearm manufacturers are held responsible by financial markets for manufacturing the very products used in these incidents. This broad question: how financial markets respond to these events in terms of the stock prices of firearm manufacturers, forms the core of the research reported in this paper.

While gun violence within the US occurs in many disparate forms, the most distressing and visible form of such violence are mass shootings. Defined by the Federal Bureau of Investigation (FBI) as any incident where at least four persons are killed with a firearm in a random act with little or no premeditation [5], these events can occur in private homes, in the shooter’s current or prior workplace, and even in schools and universities. Names such as Columbine and Sandy Hook have become cultural markers in the US, capturing the essence of the randomness of such events. The specific form of weapon used in these events also varies; Mayors Against Illegal Guns [5] report that approximate 15% of shootings between 2009 and 2013 were with automatic weapons, resulting in significantly more deaths compared to crimes where handguns are used exclusively. Finally, in 43% of such incidents, the perpetrator ended up committing suicide, further implicating the role of mental health as a critical factor.

While the details of each event differ, all represent seemingly senseless acts of violence where close family members, co-workers, and even relative strangers were adversely affected through the use of firearms. As firearms are centrally implicated in these events, extant theory would suggest two potential responses. On the one hand, considerable anecdotal data, and some recent research, shows a sharp spike in firearm purchasing immediately after such events; suggesting an arming of individuals [6, 7]. The *Financial Times of London*, for example, reported that gun sales jumped 52% in the year after Newtown, CT [8]. Alternatively, mass shootings increase the calls for additional gun control, regulation, mental health checks, and gun registration [9]. For instance, the 2014 mass shooting in Santa Barbara, CA spurred calls for greater gun control by parents of the victims, thereby making them highly salient [10]. The effects of such regulations, pending their legislative enactment, would likely introduce significant controls and restrictions on the sales of firearms in the US, thereby undermining firm profitability in the long term. This dichotomy of responses to these shootings illuminates a central tension about how the market may respond to manufacturers of firearms.

Beyond the immediate effect of mass shootings on the stock prices of firearms manufacturers, there are further likely to be moderating effects to the long-term valuations of firearms firms based on the circumstances surrounding the event. Events like Newtown and Columbine struck a chord with society as the victims included children. Similarly, the use of automatic weapons and handguns has raised the profile of certain mass shootings [11]. Thus, as an extension of our base research question, we also consider how specific circumstances about the event, such as the victims including children, the use of handguns, and the location of the shooting, affect market response.

We address these questions using a traditional market movement model event study [12, 13], a widely accepted approach in finance to model abnormal returns in the wake of exogenous events. As, almost by definition, mass shootings are random, there is perfect identification in terms of the effects of the event on a focal firm’s stock price; notably in the short-run. We use the dataset compiled by the Mayors Against Illegal Guns [5] wherein every mass shooting in the US, defined as events where at least four persons were killed, and its associated circumstances have been identified. This dataset covers the period between January 2009 and September 2013 and includes 93 events. Most firearm manufacturers in the US are private firms but two firms are public and form the focus of this study–Smith and Wesson (SWHC), recently rebranded as American Outdoor Brands, and Sturm, Ruger, and Co. (RGR). We use firm-level stock data from the Center for Research in Security Prices (CRSP) US Stock Prices dataset to identify stock price movements associated with mass shootings.

Our results indicate a significant decrease in the stock price of firearm manufacturers over a 2, 5, and 10-day window. Interestingly, we see no significant decrease in the stocks of related firms, i.e. those firms that are viewed to be in the same industry category or closely associated with these two firearm firms [14], thereby speaking to the robustness of the results and the exclusivity of the penalty to firearm manufacturers. In further analyses, we find that the penalty firearm manufacturers receive in the short-term from the market is intensified by the number of people slain and the use of a handgun. Interestingly however, we see no moderating effect if the victims are children or if the crime occurs in a conservative or liberal state, thereby underscoring the notion that the dominant mechanism by which this penalty occurs may be the risk of increased regulation. Finally, we see that market responses to mass shootings appear to taper off towards the latter half of our panel, suggesting that the market may be accepting this as the “new normal,” and not expecting any regulatory action that may affect the financial value of the firearms firms.

In terms of economic impact, results show that each mass shooting leads to a penalty of between 22.4 and 49.5 basis points, per day, imposed on firearms stocks. With the market capitalization of Smith & Wesson (Ruger), at roughly $509.11mm ($677.65mm) on January 1, 2015, this translates to a $5mm ($6.7mm) equity loss over two days alone. While these drops in stock price do not manifest in the long term, i.e. movement is not significantly different from the market after a 10-day window, analysis shows that in the immediate aftermath of the event, there is a significant drop in price. Given that firearms stocks are generally volatile, the unpredictable shock of a mass shooting viewed over a 5-day period levied roughly a 1.25% negative effect on the price on average, representing almost half of the daily volatility.

Our work makes several contributions to extant literature. First and foremost, a large corpus of research surrounding mass shootings and gun control has focused, rightfully, on the public policy, medical, legal, and sociological ramifications of gun violence in American society [1, 2, 15]. Inasmuch as the stock market and financial investors represent part of the social structures in which guns are embedded, we argue that it is also important to understand how the market responds to such events [16]. Second, we contribute to the extant literature on stigma and corporate responsibility associated with industries or firms [17]. Current work in stigma addresses the extent to which media influences stigmatized industries such as the armaments industry [18] or the extent to which wrong-doing by one firm stigmatizes other similar firms [19]. In comparison, we study a context where an industry is affected by a series of negative events that are, to a greater extent, out of its control, but are intimately associated with the industries’ core products. However, we also see that even during the relatively short period of our study (4 years), there is increasing lack of response to these events as markets appear to learn that no regulatory action is likely to be forthcoming. Thus, viewed differently, our study also documents how a stigmatized industry may actually be able to outlast negative events associated with its products, with implications for other industries such as gaming, alcohol, and tobacco [20] that may share some of these characteristics.

## Related literature

Mass shootings have been the subject of considerable research in the literature on criminology, health policy, and economics. Within each stream of research, the focus has differed in terms of the questions asked. Within criminology, the relevant questions posed pertain to the role that handguns play [21] as well as the extent to which the right to conceal arms can contribute to such events [22]. Within public health, the antecedents of mass shootings have been studied, such as the role of mental health [23] as well as the impact of media and censorship [1, 24]. Research in healthcare has also addressed the psychological consequences of mass shootings: such as the potential subsequent spikes in the suicide rate based on the easy availability of firearms [25], as well as the impact on psychiatric disorders like depression, post-traumatic stress disorder, and drug addiction [26]. In this vein, received research has widely observed that the most common method of suicide in both Australia and the United States comes from firearms [27, 28], both of which can be lowered by reducing access to firearms. Further, state-level ownership of firearms, and the resulting consequences, appears to be particularly inimical for women; Siegel and Rothman [29], for example, show how increases in firearm ownership is linked with increases in the rate of femicide. Finally, in the public health literature, considerable work has addressed the twin questions of how to curb gun-related violence as well as to how to manage its aftermath within the communities where they occur [30].

Within the domain of economics, the most closely related to the work presented here, relatively little work has studied the implications of mass shootings. Cross and Pruitt [16], who examine how the Aurora CO and Newton CT mass shootings affect the stock prices of firearm firms as well as those of the theatre company (Cinemark) where the shooting occurred, represents the nearest analog. In other work, scholars have addressed how policy changes in the right to carry concealed guns has led to spillover effects in neighborhoods [31] as well as the role of assault weapons [32]. The relationship between mass shootings and the resulting increase in demand for guns has also been studied [6], as has the impact of elections and the associated fear of impending regulations on the demand for guns [33]. Yet, within this stream there is a significant gap in our understanding of how mass shootings directly affect the response of the financial markets, i.e. the stock prices of firearm manufacturers. Further, we have limited visibility into whether these effects are moderated by characteristics of the event. This forms of the core of the work we present here.

Why would a seemingly random event such as a mass shooting affect the stock price of a firearm manufacturer? We explore arguments for why mass shootings may positively affect stock prices, through increased short-term demand for guns, as well as why the effects may be negative, through increased stigma and associated threats of regulation. It is this tension between the competing explanations that we address through our empirical analysis.

We first identify sources of ownership for firearms stocks; which is relevant to any discussion of mass shootings and financial markets. Within the United States, only two firearm firms are publicly traded (*Sturm*, *Ruger*, *and Company* and *Smith and Wesson Holding Company*, as shown in Table 1). Others are privately held. It should be noted that Smith and Wesson Holding Company rebranded itself as American Outdoors Brands Corp. (AOBC) on January 3, 2017. A third firm, Vista Outdoor, also manufactures firearms (amongst other products) but was incorporated in 2015 as a spin-off of Alliant Techsystems, an ammunition maker. The firearms division was earlier called Savage Sports Corporation, a private firm. The publicly held firms are largely controlled by institutional investors and mutual funds, such as BlackRock, Fidelity Investments, and Vanguard [34]; and are often included in retirement / pension funds (e.g. California Public Employees’ Retirement System (CalPers)). In addition, it is not uncommon for these stocks to be held by executives and insiders (Table 1). While investors may hold these assets in the long term, socially conscious investors may also choose to either divest these stocks under certain conditions or not purchase them at all. Indeed, even investment managers recognize that some of these stocks may not be attractive to all investors and thereby offer alternative vehicles such as the Vanguard FTSE Social Index Fund [34] that excludes investments in firearms, tobacco, alcohol, and gambling firms. However, the marginal decision to divest such investments may also be influenced by events outside the firm’s control. One such category of events that is likely to have an effect on investor behavior is, of course, mass shootings, where the firm’s products are utilized.

**Table 1:** Company information.

	Sturm, Ruger & Company (RGR)	Smith & Wesson (SWHC)
SIC Code	3480 (Ordnance and Accessories)	3480 (Ordnance and Accessories)
Founded	1949, Southport CT	1852, Springfield MA
No. of Employees (2012)	1460	1475
Revenues (2012)	$491.82M	$412M
Cost of Goods Sold (2012)	$312.87M	$281.5M
EBITDA (2012)	$125.93M	$64.65M
Stock Prices in 2012 (Jan–Dec)	$34.14 - $45.40	$4.49 - $8.44
Institutional Ownership of Stock (2012)	92.83%	72.64%
Institutional Ownership (2012)	17.64M	40.94M
Insider Ownership of Stock (2012)	4.24%	8.76%
Insider Ownership (2012)	0.8M	5.66M
Top 5 Stock Holders	Blackrock Fund Advisors Vanguard Group London Co. of Virginia Capital Research and Management Dimensional Fund Advisors LP	Vanguard Group Blackrock Fund Advisors Dimensional Fund Advisors LP Arrowstreet Capital Quantitative Mgmt Associates
Popular Brands	Ruger Rimfire, Centerfire, Mark II and III, Charger	Smith and Wesson, M&P, Thompson / Center Arms

Anecdotal evidence for why an investor may find firearms stocks attractive, because of a mass shooting, is available in a variety of forms. Significant evidence in the popular press attests to the notion that gun sales increase significantly after a mass shooting [35, 36]. While, traditionally, data on the actual link between mass shootings and gun sales has been difficult to come by, interviews with firearm stores and firearm enthusiasts indicate this to be true. More recently, empirical research has further corroborated the observation that mass shootings appear to be linked to a significant increase in the applications for firearms licenses [6], thereby, potentially, leading to greater firearm sales. Thus, as a first-order effect, mass shootings appear to increase firearm sales, thereby leading to higher valuation for the firms that manufacture them.

Two plausible explanations exist as to why mass shootings might lead to increased short-term sales. First, a large section of the public in the U.S. views gun ownership as a first step towards protection from crime [2, 37]. In systematic surveys, Barry et al [23] provide a more nuanced view on public opinion; namely that general gun owners like National Rifle Association (NRA) members are more tolerant of guns in society and express the sentiment that guns are useful as protective devices. Non-gun owners diverge sharply, often expressing the need for greater gun control and laws, such as banning the use of automatic weapons for recreational purposes. These differences notwithstanding, surveys by the Pew Research Center [38] and the *Economist* [39] suggest that, in the aggregate, there appears to be little change in the overall opinion across the country even after mass shootings. Therefore, for gun buyers and members of shooting associations who believe in the value of firearms for protection, the salience provided by events such as mass shootings are likely to lead to greater demand for firearms in the short-run.

Beyond protection, a second force also plays a role in driving demand; the enhanced probability of impending gun regulation. Anecdotal evidence from one firearm retailer in Virginia states as much: "*Normally what happens—and I've been doing this for 30 years–is whenever they start talking about gun control on the news and they start pushing that*, *people have a tendency to think they're going to take away their right to buy the gun*, *and that usually spurs sales* [37].” Prior work in psychology further corroborates this argument, suggesting that the effects of mass shootings can unduly influence opinions among viewers towards additional gun control; as well as restrictions on the availability of guns for people with mental health issues [40]. Thus, in the immediate aftermath of a mass shooting, there is an appearance of a groundswell movement towards firearm regulation, with a short-term effect on increased sales of firearms in the short to medium term. Given these two mechanisms, it is reasonable to expect firearm manufacturers to see significant financial benefits through increased firearm purchases, increasing their value to investors and leading to higher stock prices in the short term.

From a firm value perspective, the increased possibility of firearm sales would lead to an enhanced valuation of the firm. In such cases, we would expect an increasing number of fund managers to increase their holdings in such stocks, thereby driving up demand for firearm stock. Since short-term supply is relatively fixed, this should lead to a short-term increase in the price of the firearm stock, leading to a new equilibrium price. Anecdotal evidence for such effects can be observed, both from individual investors [34] as well as algorithmically traded funds [41]. Further, the improved outlook for firm revenues in the wake of a mass shooting may reduce the incentive for fund managers to divest from such stocks, even if individual customers are unwilling to carry them. For example, the New York City Retirement System retained ownership in firearm stocks despite recent calls by the New York City Mayor Bill de Blasio called for their divestment [42]. Thus, via the individual decisions by fund managers to increase holdings, as well as an increasing reluctance to divest, a positive short-term effect of mass shootings may manifest, pushing prices up to a new equilibrium price.

However, there is also reason to believe that markets may react negatively to such events. Mass shootings enhance the impression that firearm manufacturers bear responsibility for these events, even if they are not directly responsibility for the shootings. And, as discussed above, institutional investors may hold firms culpable for such events. Anecdotal evidence, for example, exists for how investors or classes of investors may negatively respond through their investment. The CalPers organization, for example, decided to divest such holdings as a direct response to the Newtown, CT shooting at Sandy Hook in 2013 [43]. Although firearm advocates consistently argue that many factors may be at play in such events, and that the firearms industry should not be singly implicated, such impressions do exist [23]. Further, as outlined above, there is an expectation of enhanced firearm control emerging from events of gun violence, even though manufacturers offer their products legally and bear no legal culpability for such events. This expectation of increased regulatory control of what is arguably a legal and legitimate product can be explained by prior work in stakeholder theory and social contracts.

Stakeholder theory argues that the stakeholders of the firm are not simply stockholders, but all entities within society who are directly or indirectly affected by the actions of the firm [44, 45]. While such an expanded view may not sit well with an economic view of the firm, where the stakeholders represent only ownership [46], it is necessary for the firm and its managers to construct value propositions for the firm within the broader social ecosystem [47]. Thus, part of the manager’s job is to identify conflicts and tradeoffs between diverse groups of stakeholders and to resolve these in such a way that value is maintained [48]. Further, ignoring certain stakeholders, and their legitimate claims (such as potential employees or potential customers), can have adverse effects for the firm [45, 49] (see Parmar [44] for a comprehensive review). Social contracts, based on relevant ethical standards of society, are the guidelines that managers rely on to make such tradeoffs [50], making them the informal and formal guidelines that organizations use in establishing and governing their relationships with the community [51].

When firms are viewed to be non-compliant with social contracts, such as when they contribute directly or indirectly to violence, communities and social entities have the means to punish the firm appropriately [45]. Such punitive actions include being deprived of business opportunities or partnerships [50], imposition of higher transaction costs on deviant firms, boycotts by customers, and regulatory action [51]. The stigma attached to the financial services industry after the 2008 financial crash [52], for example, or the accounting scandals from the early 2000s are instances of industries which were socially and legally sanctioned [53], even though not every action taken by these firms represented a legal breach.

Applying this lens to mass shootings, we argue that mass shootings may lead to an increased perception amongst stakeholders that firearm firms are in breach of their social contract. While firearms are legal products, unethical marketing and sales practices have also been noted within the industry in the US, making access to firearms easier than it should be [54]. Moreover, easily available protective devices for firearms (such as trigger locks), though available, have often been underutilized by manufacturers, primarily for financial reasons. Green [54] writes: “Too often, instead of showing leadership in improving their products or practices and positioning their industry for long-term viability, business owners and managers have merely resisted change. Eventually, bad practices undermine the conditions for continued growth or invite heavy-handed government regulation” (p.204). Mass shootings may focus attention on the part of the market on these sectors where firearms firms may be viewed as not adhering to their social contract, leading to the notion of “moral responsibility” increasingly being attached to them as a result of mass shootings [55]. The results of these “breaches” in the social contract are thus likely to result in an increased perception of impending sanctions, in the form of regulations. Regulations in this context are linked to reducing the long-term valuations of the firm, and therefore negative stock market responses.

Indeed, even in the absence of explicit regulation, perceptions of wrong-doing (even indirect) can result in stigma accruing to firearm stocks, leading many investors associated with specific pension funds, endowments, religious organizations, banks, and insurance companies, to divest the stock [20]. Beyond firearm purchases, anecdotal evidence also exists for how investors or classes of investors may choose to respond through their investment decisions. The CalPers organization, referenced above, decided to divest its investments in two firearm makers as a response to the Newtown CT mass shooting in 2013. As organizations and investors decide to divest stock, they create increased liquidity within the market, signaling that the stock may be over-valued [56]. These dynamics may then lead to increased selling by other investors, resulting in downward price pressure. This downward pressure will continue until the stock price reaches a new equilibrium that is lower, as the firm fundamentals are likely viewed as being weaker. In summary, these mechanisms suggest a negative relationship between a mass shooting and the short-run stock price movement.

In summary, while we provide arguments for why mass shootings may result in abnormal stock movements for firearms manufacturers in the US, whether the net response from the market is positive or negative remains an empirical exercise. While there is anecdotal evidence for such effects, empirical research in this domain is lacking. In the following section, we first provide model-free evidence to illustrate how the perception of regulation and gun control may be driving market responses to mass shootings, and subsequently delve into the empirical analysis to examine this question rigorously.

## Empirical analysis

### Model-free evidence–google trends and mass shootings

Before we conduct our econometric investigation into how mass shootings may affect stock price, it is useful to understand how public discourse may be affected by mass shootings. One of the commonly expected effects of mass shootings is an increase in the firearm license applications, which may viewed as a precursor to higher demand for firearms, and thereby lead to higher stock prices. Indeed, Price [57] shows such an effect in license application data. Further, as one of the common responses to mass shootings appears to be an increased expectation of regulation, one plausible first order check is an uptick in discourse regarding the regulation of firearms. Put another way, the increased salience of gun control and regulation that follows such an event may lead to increased Internet searches for associated terms. Such methods have been used in prior research to understand market and political trends [58]. Google search trends have also been used to capture trends relating to consumer confidence, real estate prices, and trading behavior on stock exchanges [58–60]. While Internet searches do not meet the requirements of econometric identification, given the many confounding factors that may drive search behavior on the Internet, they do allows us to provide model-free evidence of increased salience, thereby establishing some veracity to the argument that mass shootings may influence the expectations of regulatory action.

Adopting this approach, we capture Google search trends immediately following mass shootings using the following terms: “gun control”, “gun laws”, “handguns”, “NRA”, and “mass shooting”. Google search trends represent the relative proportion of all Internet searches conducted on Google that pertain to the specific terms included in the query; therefore, they provide a normalized measure of the relative increase or decrease of Internet searches associated with the specified terms over time. Typically, these trends correlate highly with underlying offline or online events, thereby serving as a reasonable reflection of sentiment or activity online [59]. We select the above-mentioned terms since they are associated with both the role of firearms and the possibility of gun-related regulation within public discourse. Since we expect that mass shootings are likely to enhance expectations of regulations, it would follow that the search trends for terms such as “gun control”, “gun laws”, and “handguns” would see a significant increase. A similar trend would also be expected for “NRA.”

**Fig 1:**
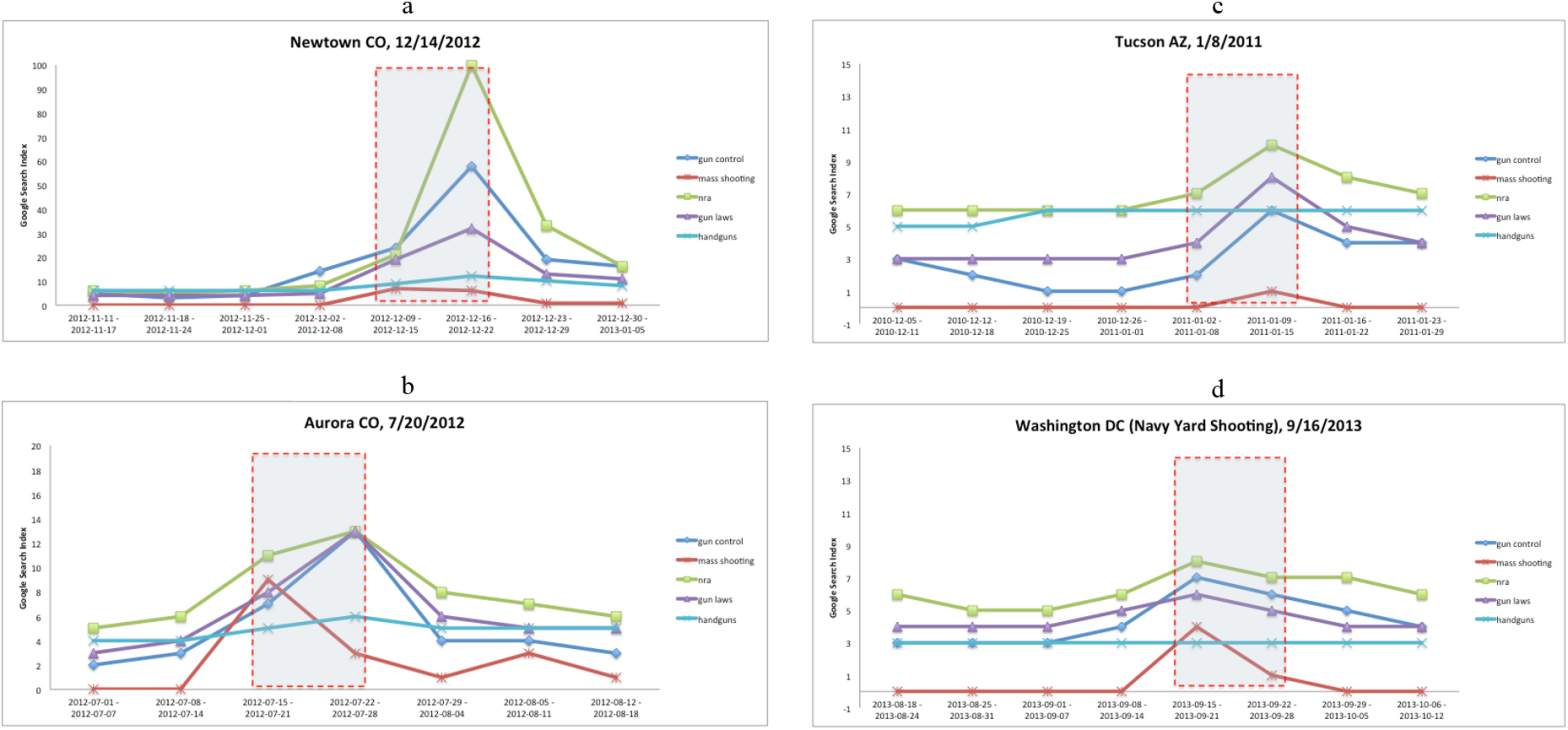
Google search trends for keywords associated with mass shootings.

We plot the Google search trends for four highly visible and salient mass shooting events as part of this exploratory analysis. Note that Google releases trends on a weekly basis. Therefore, we cannot identify search spikes relative to the day of the mass shooting. Fig 1A–1D show plots for following mass shooting events: Newtown CT (December 14, 2012), Aurora CO (July 20, 2012), Tucson AZ (January 8, 2011), and the Washington DC Navy Yard shooting (September 16, 2013). These weekly data plot the 3–4 weeks prior to the event, the week of the event, as well as 2 weeks post event. The shaded box is a rough estimate of the 10-day interval following each event. In each of these cases, a clear increase in search trends for “gun control”, “gun laws” and “NRA” can be seen, while the trends for “mass shootings” are less compelling. The Y-axis captures the relative share of the search term in searches conducted during the period. Clearly, search behavior during the period surrounding the Newtown CT event was dominated by the event, given its particularly high salience. In other cases, the relative spike in search is less striking, but discernible nevertheless. While this analysis is not statistical in nature, it lends support to our thesis that the threat of regulation does increase in the aftermath of a shootings. In the next section, we describe the statistical analysis in detail.

### Data and estimation procedure

**Fig 2:**
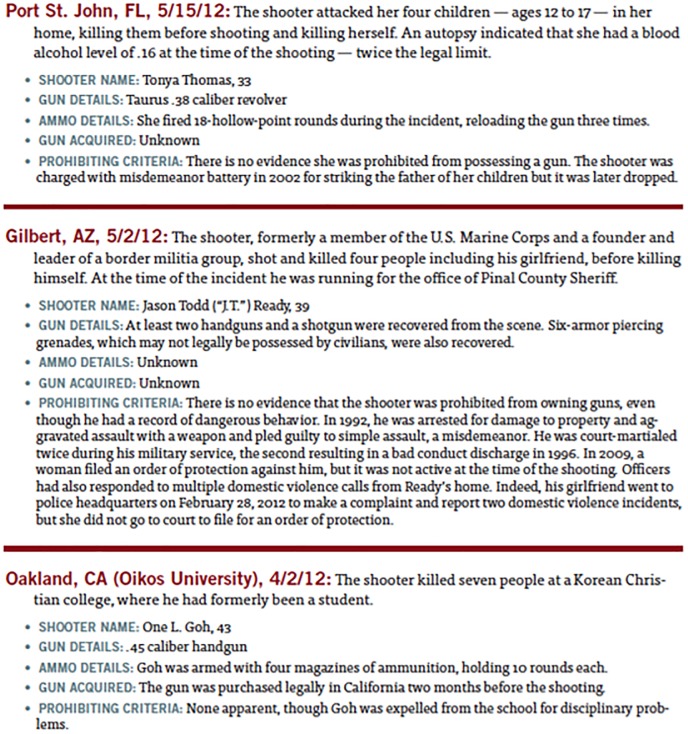
Snapshot of the mass shootings dataset.

In this analysis, our main objective is to identify the effects of mass shootings on the short-term movement of firearm stock prices. To that end, we create a dataset drawing from multiple publicly available sources of data. First, to capture the stock prices of existing firms we extract the relevant stock data from the Center for Research in Security Prices (CRSP) US Stock Database. CRSP is currently the largest and most comprehensive historical stock market dataset in the world; and has been used extensively in prior work. This gives us the daily stock price of every publicly traded firm in the US, thereby allowing us to calculate both the movement of firearm manufacturers as well as other firms in the market. Data on mass shooting is drawn from Mayors Against Illegal Guns [5] press releases. Spearheaded by former New York City and Boston mayors Michael Bloomberg and Thomas M Menino, this rich source of data grants us access not only to the date of shootings, but the number of people affected, location, and whether or not a handgun was involved (see Fig 2). Between 2009 and 2013, 93 individual incidents were recorded that qualified under the definition of a mass shooting, including the four described above in the Google Trends analysis. The information on these events is extracted from the reports and coded appropriately. For the baseline analysis, we only use the date of the event to study the market’s response. Descriptive statistics of the 93 events can be found in Table 2.

**Table 2:** Summary statistics.

		Mean	Std. Dev.	(1)	(2)	(3)	(4)	(5)
1	r	0.0009199	0.0350313					
2	Market	0.0002489	0.017966	0.4811				
3	NumKilled	5.26087	3.040239	-0.062	0.1011			
4	Children	0.3913043	0.4893739	-0.0734	0.0112	-0.069		
5	Handgun	0.6847826	0.4658701	-0.0003	-0.0508	0.1818	-0.1751	
6	Cook PVI	-1.206522	10.76885	-0.0571	-0.0469	0.1876	-0.2127	-0.0239

N– 93 Mass Shootings.

The primary empirical technique we employ is a market movement event study methodology [12, 13], a well-accepted method in finance [61] and economics [62, 63] to study market responses to new information made available through selected firms or specific events. Using this methodology, we study the abnormal returns which accrue to firearm manufacturers after a mass shooting over a one, two, five, and ten-day window for the 93 mass shootings. Accordingly, the dependent variable for this investigation is the *daily percent change in the stock price* (*r*) of the two publicly held firearm manufacturers: Smith & Wesson (SWHC) and Sturm, Ruger, & Co. (RGR). Following the standard event study methodology, we regress *r* on the percent change in return, compared to an appropriate market index, over the window of the regression (1, 2, 5, and 10 days before and after treatment). The treatment, i.e. the mass shooting, is dichotomous and applied on the first full day of trading after the event occurs to prevent partial information dissemination (i.e. the shooting occurring after trading has closed or traders not being fully informed of the event) from contaminating the empirical analysis.

Utilization of a market movement event study offers us several significant advantages. First, to the extent that the dependent variable is the percent change in stock price of firm *i*, we mitigate concerns about serial correlation of the error terms because the change not attributable to the treatment is assumed to be a random walk, following the efficient markets hypothesis [64]. Second, as the shootings themselves are exogenous and unpredictable events, and the effect is measured in the short term, we need not include additional covariates to account for the propensity for the stock to over or under perform against the market in the long term, e.g. Fama-French factors [65]. More simply, because the treatment can be assumed to be exogenous, we need only determine how the stock price of firm *i* generally trends with the market (in effect, “training” the model). The choice of an appropriate market index is critical to create a suitable set of firms that can be used as counterfactuals. Consistent with the literature, we consider two specific sets of firms to account for market movement. The first market index we use is the Standard and Poors’ (S&P) 500 Market Index, which captures the broad movement of influential firms in the market. We thus include the appropriate % change in the S&P 500 market return during day *j*. However, prior research in finance has also argued that a characteristic-based matching approach provides a closer match to focal firms that using a broad market index such as the S&P [66, 67]. Within this approach, the focal firm (*SWHC* or *RGR* here) is matched to a smaller set of firms that are a closer match in terms of three specific characteristics–average size, book to market, and stock momentum. Based on these characteristics, the firm can be allocated to a specific portfolio of stocks, which then form the appropriate “market.” Following Daniel et al [66], we use this methodology and control for the appropriate % change in the equally-weighted portfolio of stocks during day *j* (referred to as the *DGTW market index*). Note that the re-allocations of the portfolios to individual firms are conducted on an annual basis for this dataset, and is only available until 2010 (alex2.umd.edu/wermers/ftpsite/Dgtw/coverpage.htm). Therefore, for this analysis, we utilize the portfolio allocation from 2010 for the remaining years of our dataset.

Finally, it is important to note that choosing the window of investigation for event studies represents an inherent trade-off. A shorter window may not allow the model to sufficiently capture how the market and the return to the stock price of firm *i* correlate, and too long a window allows for other events to contaminate the analysis, thereby creating identification problems. To remedy this concern, we estimate the effect over many different windows, while keeping the length of the window constant before and after the event. We therefore estimate the effect of mass shootings on firearm manufacturer stock price using the following equation and an OLS estimator:

$$r_{ij}=\beta_{1}x_{1}+\beta_{2}{Market}_{j}+\alpha +\varepsilon_{ij}$$

where *r* represents return for firm *i* on day *j*, *x* is the dichotomous treatment indicator, and *Market* is the change in S&P 500 or DGTW market movement on day *j*. α and ε represent the constant and error terms, respectively, and {β_1_, β_2_} represent the parameters to be estimated. Intuitively, the estimated model represents a differences-in-differences approach wherein the stock price variation of firearm manufacturers is compared to the average movement of the stock prices of the S&P 500 Index / DGTW Index before and after the event, within the stipulated time window. *x* should therefore be interpreted as the percent change, per day, post treatment. Moreover, since the shootings are random, comparing stock prices before and after the event allows a clean identification of the effect of the shooting event on firearm manufacturers. Widening the time window of the analysis allows for the possibility of confounding events. However, within the shorter time windows, the effects of the mass shootings on firearm manufacturer stock can be identified cleanly and without bias. We bootstrap standard errors in order to obviate concerns of finite sample bias, as well as parametric assumptions about the functional form of the standard errors themselves [68]. Results are in Tables 3 and 4.

**Table 3:** Effect of mass shootings on firearm manufacturer market returns.

	(1)	(2)	(3)	(4)
Dependent Variable	r	r	r	r
Time Window	1 Day	2 Day	5 Day	10 Day
Treatment	-0.00265	-0.00495**	-0.00279*	-0.00243**
	(0.00267)	(0.00247)	(0.00160)	(0.00114)
Market Return	1.047***	1.076***	1.051***	1.100***
	(0.130)	(0.102)	(0.0779)	(0.0603)
Constant	0.00326*	0.00322**	0.00171	0.00284***
	(0.00182)	(0.00127)	(0.00110)	(0.000709)
N	552	920	2,024	3,864
R-squared	0.188	0.188	0.187	0.199

Dependent Variable: Percent Change in Firm Stock Price.

Model: OLS Market Movement Event Study.

Bootstrapped standard errors in parentheses.

*** p<0.01

** p<0.05

* p<0.1.

**Table 4:** Effect of mass shootings on firearm manufacturer market returns.

	(1)	(2)	(3)	(4)
Dependent Variable	r	r	r	r
Time Window	1 Day	2 Day	5 Day	10 Day
Treatment	-0.00159	-0.00513**	-0.00237	-0.00215*
	(0.00316)	(0.00210)	(0.00156)	(0.00110)
DGTW Return	0.264***	0.275***	0.237***	0.231***
	(0.0413)	(0.0336)	(0.0247)	(0.0185)
Constant	0.0161***	0.0174***	0.0140***	0.0145***
	(0.00246)	(0.00223)	(0.00158)	(0.00110)
N	552	920	2,024	3,864
R-squared	0.108	0.121	0.088	0.087

Dependent Variable: Percent Change in Firm Stock Price.

Model: OLS Market Movement Event Study.

Market Controlled for using DGTW.

Bootstrapped standard errors in parentheses.

*** p<0.01

** p<0.05

* p<0.1.

## Results

Results in Tables 3 and 4 and provide several interesting observations. We first address the results pertaining to the S&P 500 market index. The coefficients for the Treatment variable represent the abnormal day-specific returns to the mass shooting, relative to the pre-event correlation of the stock with the S&P 500 market index. First, as expected, we see that single day market return is strongly correlated with the S&P 500 market index movement, indicating that the stock prices of these firms tends to track the market well. Furthermore, although we see no significant effect in the first day, post shooting, we see a significant drop in the stock prices of firearm manufacturers between 49.5 and 22.2 basis points, per day, over a 2, 5, and 10 day window. With the market capitalization of Smith & Wesson (Ruger), at roughly $509.11mm ($677.65mm) on January 1, 2015, this translates to a $5mm ($6.7mm) equity loss over two days alone, providing a clearly identifiable economic impact.

Moving to the results with respect to the DGTW market index, we see broadly similar patterns in the results, with a few caveats. First, unlike the S&P index, the base correlation between the DGTW market index and the stock prices of the firearm firms is significantly lower (0.264, in the 1-day model), suggesting that the DGTW index may have weaker association with firearm stocks than the S&P. Further, we see lower R^2^ values associated with these analyses, again relative to the S&P 500, suggesting that the S&P Index may be a stronger counterfactual. One plausible explanation for this decreased explanatory power is the portfolio allocations; recall that data limitations do not allow us to modify the characteristic-based portfolios for the firearm firms post 2010. Therefore, to the extent that the specific characteristics between the two firearm firms and their matched portfolio firms diverge in subsequent years of our panel, the ability of the DGTW market index to provide a reasonable counterfactual will suffer. Regardless, we see a significant effect of the event treatment on the two- day market return, with a coefficient value that is similar to that provided by the S&P market index. The 5-day and 10-day coefficients are also similar in magnitude, though the statistical significance is considerably reduced.

During the period of our analysis (2009–2013), the volatility of the firearms stock was reasonably high. For instance, the volatility of Smith and Wesson (SWHC) stock was roughly 41%, which devolves to a daily volatility of roughly 2.45%, representing variability that is roughly structural. The *unpredictable* effect of a single mass shooting in our model on SWHC stock over a 5-day period, using the S&P market index, is roughly 25 basis points per day, which amounts to a total effect of 1.25% negative shock on the price. In terms of magnitude, this is approximately half of the daily volatility, suggesting a substantial economic impact. A similar calculation for Ruger stock shows the same qualitative outcome. Clearly, mass shootings contribute significantly to the volatility observed in firearms stock, except that these movements are all uniformly negative and unpredictable.

A further test for the effects of mass shootings on firearm firms can be conducted by comparing the effects from Tables 3 and 4 to a set of firms that are associated with firearm firms but do not manufacture firearms directly. These firms can be found in the network of firms that form the extended value chain for RGR and SWHC, such as retailers who sell firearms, for instance, or steel firms that supply raw materials. When a negative event, such as an industrial accident, is reported, associations with the errant firm often spread to other firms or industries that are viewed as being similar [19]. Similarity, the effects here may involve other firms in the value chain, customers or suppliers of the focal firm or industry, or in some extreme cases, other firms with subjective impressions of “likeness” [18]. Therefore, it is possible that these associated firms are also affected by mass shootings. However, if the mass shooting’s effect is limited only to the firearm firms, it is reasonable to conclude that the stock price effects identified are more pertinent to the shooting event, and not part of a larger industry-wide or economy-wide shock. Such a test thus operates as a robustness test for the effect of mass shootings on the two firearm manufacturers.

We define similarity of other firms with the two firearm manufacturers using recent work in finance based on text analysis of firm 10K filings, the Text-based Network Industry Classification (TNIC) [14, 69]. This process dynamically matches all public firms with other firms using text analysis of the firm’s 10k filings and creates similarity indices between firms which can be established and modified year-on-year (see Hoberg and Phillips [14] for a complete description of this methodology). Hoberg and Phillips [14] thus offer a scheme that allows us to identify all firms in a year that are “similar” to the two firearm manufacturers based on textual analysis of their respective 10k filings (further details on this methodology are provided in the Statistical Appendix and comprehensive data is available at: http://hobergphillips.usc.edu/). We therefore replace the two firearm firms in the baseline model with the firms in their respective TNICs, and re-estimate the market movement model using the S&P market index. The treated firms here are those firms that are similar to the two firearm firms.

Results in Table 5 show no significant effects of the treatment, indicating that the observed negative effects from above are specific to the two firearm firms and not extended to other “similar” firms. This result adds some support to the notion that the perception of regulation may be responsible for the negative effect on firearm stocks. Any such regulatory effects may affect other public firms that are part of the ecosystem but not as directly as they will affect firearm manufacturers. For instance, retailers like Walmart may not be as directly affected by impending regulation, even though firearms constitute a non-trivial share of revenues [70].

**Table 5:** Effect of mass shootings on TNIC market returns.

	(1)	(2)	(3)	(4)
Dependent Variable	r	r	r	r
Time Window	1 Day	2 Day	5 Day	10 Day
Treatment	0.00139	-5.68e-05	-0.000433	0.000226
	(0.00335)	(0.00197)	(0.00116)	(0.000880)
Market Return	1.189***	1.103***	1.096***	1.049***
	(0.111)	(0.0815)	(0.0521)	(0.0431)
Constant	0.00153	0.00149	0.00135*	0.000904
	(0.00146)	(0.00125)	(0.000727)	(0.000629)
N	1,104	1,840	4,048	7,728
R-squared	0.142	0.129	0.140	0.126

Dependent Variable: Percent Change in Firm Stock Price.

Bootstrapped standard errors in parentheses.

*** p<0.01

** p<0.05

* p<0.1.

### Robustness checks

While the results in Tables 3 and 4 show the cumulative abnormal returns associated with the mass shootings, it is also useful to evaluate how the event affects individual *day-level returns* (between the periods [-10..20] inclusive). To do so, we replace the dichotomous treatment indicator with a series of dummy variables to capture the average abnormal return for each day in the panel associated with the event. Intuitively, this model allows for the identification of pre-treatment trends, if any, as well as the average return by day post-event. Results from this relative time model are in Table 6, with individual columns for the S&P 500 market index, the TNIC, and the DGTW index. The results indicate no systematic pre-treatment trends in the 10 days leading up to the event. In each column, we see one or two significant coefficients in the pre-treatment period, but no systematic trend across them. Post-treatment, the S&P market index model shows significant negative effects for the first five days after the mass shooting. A similar trend of negative coefficients is obtained with the DGTW index, though only one coefficient is significant at conventional levels. The TNIC firms show no effects from the treatment, as expected from above.

**Table 6:** Relative time model of daily returns pre- and post-event using firearm manufacturers, TNIC counterfactual set, and DGTW as control for market.

	(1)	(2)	(3)
Dependent Variable	Base	TNIC	DGTW
Rel Time_t-10_	-0.00619*	-0.00151	-0.00643**
	(0.00364)	(0.00259)	(0.00312)
Rel Time_t-9_	-0.00427	-0.00185	-0.000974
	(0.00366)	(0.00272)	(0.00387)
Rel Time_t-8_	-0.00200	0.000745	-0.00324
	(0.00350)	(0.00240)	(0.00297)
Rel Time_t-7_	0.00214	-0.00420	0.00295
	(0.00385)	(0.00280)	(0.00292)
Rel Time_t-6_	0.00196	-0.00397*	0.00275
	(0.00382)	(0.00231)	(0.00386)
Rel Time_t-5_	-0.00155	-0.00281	-0.000962
	(0.00320)	(0.00236)	(0.00342)
Rel Time_t-4_	-0.00307	0.00251	-0.000255
	(0.00437)	(0.00316)	(0.00375)
Rel Time_t-3_	-0.0127***	-0.00342	-0.0123***
	(0.00342)	(0.00293)	(0.00417)
Rel Time_t-2_	-0.00287	-0.000705	0.000506
	(0.00369)	(0.00230)	(0.00305)
Rel Time_t-1_	Omitted Category		
Rel Time_0_	-0.00527*	-0.00206	-0.00424
	(0.00318)	(0.00275)	(0.00327)
Rel Time_t+1_	-0.00539	0.000726	-0.00346
	(0.00336)	(0.00342)	(0.00300)
Rel Time_t+2_	-0.00997**	-0.00255	-0.00878**
	(0.00401)	(0.00257)	(0.00354)
Rel Time_t+3_	-0.00600*	0.00195	-0.00469
	(0.00363)	(0.00300)	(0.00346)
Rel Time_t+4_	-0.00723*	-0.00289	-0.00481
	(0.00371)	(0.00267)	(0.00375)
Rel Time_t+5_	-0.00679**	-0.00445	-0.00418
	(0.00317)	(0.00283)	(0.00366)
Rel Time_t+6_	0.00270	-0.00279	0.00444
	(0.00320)	(0.00240)	(0.00385)
Rel Time_t+7_	-0.00452	-0.00132	-0.00217
	(0.00379)	(0.00263)	(0.00371)
Rel Time_t+8_	-0.00445	0.000518	-0.00348
	(0.00320)	(0.00242)	(0.00319)
Rel Time_t+9_	-0.00733**	0.000830	-0.00807**
	(0.00352)	(0.00271)	(0.00325)
Rel Time_t+10_	-0.00601*	-0.00332	-0.00607**
	(0.00363)	(0.00230)	(0.00260)
Rel Time_t+11_	-0.00540	-0.00123	-0.00460
	(0.00331)	(0.00240)	(0.00382)
Rel Time_t+12_	-0.00416	0.00103	-0.00398
	(0.00389)	(0.00269)	(0.00371)
Rel Time_t+13_	0.00219	-0.000180	0.00292
	(0.00359)	(0.00291)	(0.00356)
Rel Time_t+14_	-0.00446	-0.00351	-0.00498*
	(0.00327)	(0.00225)	(0.00282)
Rel Time_t+15_	-0.00542*	-0.00336	-0.00326
	(0.00310)	(0.00247)	(0.00320)
Rel Time_t+16_	-0.00847**	0.00149	-0.00883***
	(0.00348)	(0.00301)	(0.00296)
Rel Time_t+17_	-0.00313	-0.00310	0.000146
	(0.00345)	(0.00247)	(0.00363)
Rel Time_t+18_	-0.00362	0.00107	-0.00136
	(0.00410)	(0.00254)	(0.00351)
Rel Time_t+19_	-0.00613*	-0.00434**	-0.00537
	(0.00316)	(0.00214)	(0.00340)
Rel Time_t+20_	-0.00217	-0.000434	-6.57e-05
	(0.00398)	(0.00237)	(0.00444)
Market Return	1.093***	1.028***	
	(0.0396)	(0.0322)	
DGTW Return			0.215***
			(0.0147)
Constant	0.00592**	0.00249	0.0158***
	(0.00273)	(0.00164)	(0.00256)
N	5,646	11,292	5,646
R-squared	0.196	0.120	0.087

Bootstrapped standard errors in parentheses.

*** p<0.01

** p<0.05

* p<0.1.

**Fig 3:**
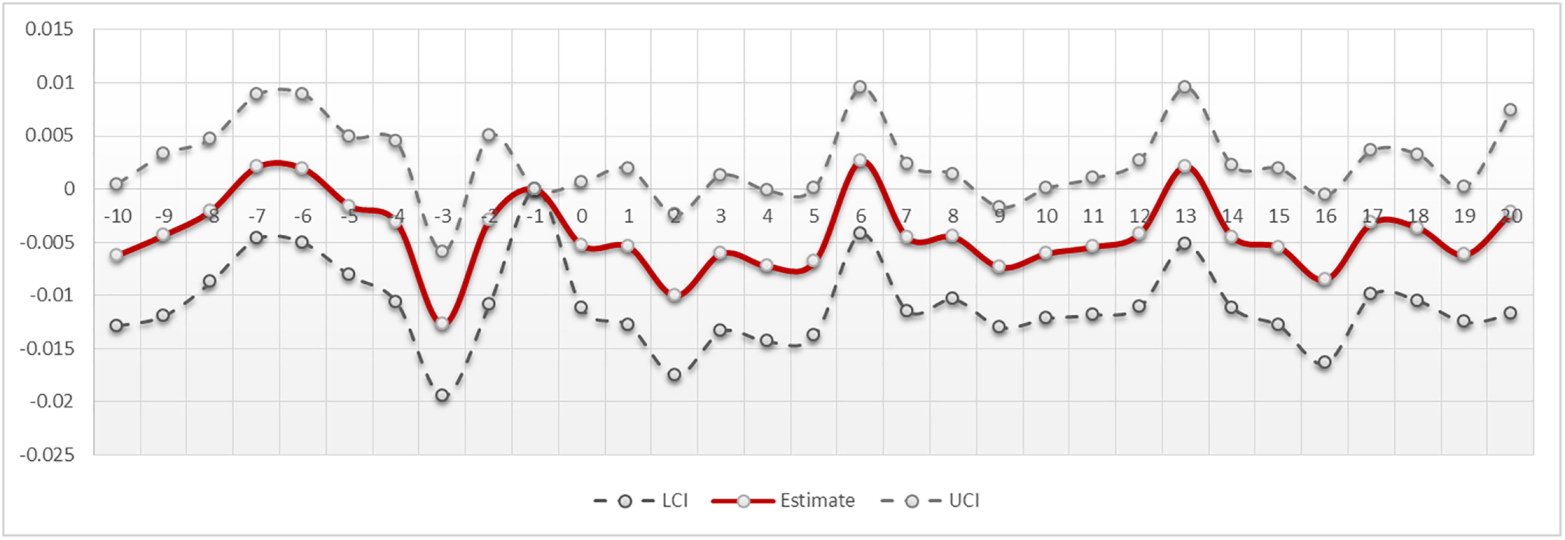
Relative time model–day-wise treatment effects. Lci And Uci indicate 95% Confidence Intervals.

These results can also be displayed graphically–Fig 3 plots the coefficients for each day in the panel data series. As is evident, there is considerable variability in the returns to the firearms stocks visible in the [-10..+20] period. However, the significant effect of the mass shooting can be discerned in the five periods after the event, highlighted in the graph. During this period, note that even the 95% upper confidence interval bound is negative, which does not occur in any other period graphed. The effect appears to be the strongest on second day post-event, consistent with the results in Tables 3 and 4. Beyond five days, we see no statistical significance to the movement of the stock prices, relative to the market. This analysis also allows us to test if the observed price pressure is temporary, consistent with the temporary price pressure hypothesis [71], wherein prices would rebound to the original prices. If demand for equity is not fully elastic, a large sale of firearm stocks would be accompanied by lower prices, which will be reversed in subsequent periods. However, we see no signs of a significant and positive effect in the days after the significant negative movement in the prices, suggesting that mass shootings appear to reflect new information about the firm, which are then incorporated into a new equilibrium price [72]. The negative effect thus lasts for five days, after which the new price is reached and any subsequent treatment effect (whether positive or negative) is absent.

We conduct several additional robustness and falsification tests of the baseline regressions to establish that the results are unambiguous. In the interest of brevity, and owing to the weaker base correlation with the DGTW index, we estimate these and all subsequent models using the S&P market index. It should be noted, however that all results are consistent with the DGTW market index. These tests include providing individual results for the two firms, modifying the training period for each model, including other placebo effects (i.e. the deaths of celebrities), as well as estimating different regression specifications for the main effects. These tests are described in the Statistical Appendix and provide largely consistent results. In the following sections, we explore possible mechanisms for why the observed negative effects of mass shootings on short-run stock prices manifest.

## Empirical extensions: In search of causal mechanisms?

The results reported above provide evidence that mass shootings negatively affect firearm stock prices. However, the characteristics of these events can be probed further to better understand the mechanism by which these events may be leading to negative pressure on firearm stock prices. In this section, we conduct a series of tests to evaluate the moderating influences of contextual variables associated with each event. These analyses allow us to test the robustness of the results, in terms of falsifiability and identifying boundary conditions, as well as establish which specific aspects of shootings may be more or less damaging to firearm manufacturers. In these tests, we use the traditional market movement model, as opposed to the relative time model, to ease with the comparison of coefficients across estimates.

### Scope and composition of victim pool

We consider the impact of the composition of the victims affected by the shooting. By definition, mass shootings lead to at least four fatalities. However, beyond this threshold, the number of victims directly influences the salience of the event within the broader environment (by virtue of media, social media, and private discourse [16]), leading to a stronger response from the market. Furthermore, we investigate if children, i.e. if anyone under the age of 18, were involved. Prior research argues that the greater the assumed level of innocence amongst the putative victims of wrongdoing, the greater is the disapproval attached to the associated firm or industry [18]. Note also that studying the number of fatalities, in addition to whether a child was involved, allows separating the scope of the shooting from the composition of the victim pool, an important distinction in the context of the possibility of regulatory action. We therefore replace the dichotomous treatment variable in our baseline analysis with the count of the victims. Additionally, to capture possible non-linear effects, we use the natural log of the victim count. Then, we split the sample into events with and without child victims and estimate split-sample analyses to evaluate the market’s response separately.

Results in Tables 7 and 8 indicate nuance in market response. First, we see a significant effect of victim count on the negative response from the market extending up to five days after the event. When using log(victim count), we see an effect extending up to two days after the event, after which the results are no longer distinguishable from market movement. Strikingly, we see no significant effect on stock prices arising from the presence of children, beyond the event itself. Aside from one coefficient, marginally significant at the (p<0.1) level, there appears to be no systematic response to children victims. A comparison of the coefficients across Table 8 using a Wald’s test following a seemingly unrelated regression (i.e. a comparison of Column 1 to Column 5, Column 2 to Column 6, and so forth) [73], confirms the lack of statistical difference across these two sets of estimates. It is further possible that the effect of victim count is curvilinear. To test this we include a quadratic term in the analysis. Results, shown in Table 9, show the presence of a curvilinear effect. While the first-order term is positive and significant in Columns (3) and (4), in each case it is dominated by the negative and significant second-order term, showing that as the number of victims increases, the punitive effect on firearm stock prices increases non-linearly.

**Table 7:** Effect of mass shootings market returns by number of victims.

	(1)	(2)	(3)	(4)	(5)	(6)	(7)	(8)
Dependent Variable	r	r	r	r	r	r	r	r
Time Window	1 Day	2 Day	5 Day	10 Day	1 Day	2 Day	5 Day	10 Day
NumKilled	-0.00126***	-0.00136***	-0.000451	-0.000204				
	(0.000320)	(0.000382)	(0.000289)	(0.000193)				
Ln(NumKilled)					-0.0108***	-0.0115***	-0.00256	-0.000518
					(0.00400)	(0.00388)	(0.00304)	(0.00181)
Market Return	1.059***	1.088***	1.051***	1.099***	1.061***	1.088***	1.050***	1.098***
	(0.130)	(0.101)	(0.0584)	(0.0500)	(0.116)	(0.0956)	(0.0581)	(0.0382)
Constant	0.00899***	0.00836***	0.00281*	0.00275**	0.0216***	0.0216***	0.00499	0.00260
	(0.00232)	(0.00204)	(0.00156)	(0.00107)	(0.00719)	(0.00683)	(0.00534)	(0.00318)
N	552	920	2,024	3,864	552	920	2,024	3,864
R-squared	0.198	0.197	0.187	0.198	0.195	0.193	0.186	0.198

Dependent Variable: Percent Change in Firm Stock Price.

Bootstrapped standard errors in parentheses.

*** p<0.01

** p<0.05

* p<0.1.

**Table 8:** Effect of mass shootings on market returns, sample split based on whether or not a child is a victim.

	(1)	(2)	(3)	(4)	(5)	(6)	(7)	(8)
Dependent Variable	r	r	r	r	r	r	r	r
Time Window	1 Day	2 Day	5 Day	10 Day	1 Day	2 Day	5 Day	10 Day
Sample	No Children				Children			
Treatment	-0.00233	-0.00369	-0.00225	-0.00313**	-0.00300	-0.00692*	-0.00364	-0.00139
	(0.00275)	(0.00266)	(0.00184)	(0.00131)	(0.00529)	(0.00397)	(0.00253)	(0.00155)
Market Return	0.955***	1.032***	1.063***	1.082***	1.181***	1.133***	1.033***	1.120***
	(0.175)	(0.129)	(0.105)	(0.0727)	(0.129)	(0.171)	(0.0984)	(0.0600)
Constant	0.00461**	0.00379**	0.00234*	0.00344***	0.00119	0.00232	0.000717	0.00194*
	(0.00199)	(0.00158)	(0.00127)	(0.000938)	(0.00273)	(0.00192)	(0.00191)	(0.00113)
N	336	560	1,232	2,352	216	360	792	1,512
R-squared	0.165	0.171	0.187	0.187	0.223	0.213	0.186	0.214

Dependent Variable: Percent Change in Firm Stock Price.

Bootstrapped standard errors in parentheses.

*** p<0.01

** p<0.05

* p<0.1.

**Table 9:** Effect of mass shootings market returns by number of victims.

	(1)	(2)	(3)	(4)
Dependent Variable	r	r	r	r
Time Window	1 Day	2 Day	5 Day	10 Day
NumKilled	-0.00407**	-0.00499**	-0.00412**	-0.00300**
	(0.00171)	(0.00214)	(0.00174)	(0.00134)
NumKilled ^ 2	0.00663	0.00858*	0.00866**	0.00660**
	(0.00428)	(0.00489)	(0.00382)	(0.00294)
Market Return	1.050***	1.080***	1.046***	1.096***
	(0.139)	(0.0994)	(0.0574)	(0.0572)
Constant	0.00228	-0.000288	-0.00591	-0.00390
	(0.00589)	(0.00524)	(0.00370)	(0.00286)
N	552	920	2,024	3,864
R-squared	0.200	0.200	0.190	0.200

Dependent Variable: Percent Change in Firm Stock Price.

Model: OLS Market Movement Event Study.

Bootstrapped standard errors in parentheses.

*** p<0.01

** p<0.05

* p<0.1.

It is surprising that the results reflect a strong effect of victim count but not the presence of children. Children are involved in fewer of the incidents but these events arguably carry tremendous salience. Though the data includes the Sandy Hook shooting in Newtown, CT, where 20 elementary school children were killed, this remains an aberration even within the context of mass shootings in the US. The presence of children as victims undoubtedly makes mass shootings salient [23], but the tepid response from the stock market when compared to the stronger response from the victim count suggests that it is the number of fatalities that is critical, not the presence of children, which may be viewed as incidental in nature. From a regulatory standpoint, the number of victims of mass shootings is relevant information (since this is a systematic outcome of any such event and can be potentially addressed through policy) but the presence of children may be viewed as less instrumental or non-diagnostic in terms of setting policy [16]. While it is impossible to conclusively determine that impending regulation is key here, we conduct further analyses to probe this eventuality. We consider the use of handguns, a clear candidate for how regulation may be driving investor behavior.

### Use of handguns

To the extent that a wide variety of firearms have been employed in mass shootings, we investigate whether or not the involvement of a handgun exacerbates or attenuates the negative effect on stock prices. We use handguns for the following reasons. While assault weapons are largely regulated by the Violent Crime Control and Law Enforcement Act of 1994, handgun regulation has, to a greater or lesser degree, become the purview of state and municipal governments. Furthermore, regulations or bans on the use of shotguns and hunting rifles are rare given their use in recreational sport (viz. hunting) and their importance in agricultural and ranching work. In contrast, handguns have seen multiple, and often failed, attempts to increase regulation at the federal level; such as the 1993 Firearms Safety and Violence Prevention Act. Further, the Federal Consumer Product Safety Commission (CPSC) does not regulate handguns as it does almost all other consumer products. Finally, the debate on the right to carry concealed handguns [21, 31] and its effect on gun violence [22] continues, making this a particularly salient factor to consider from a regulatory perspective.

To determine the effect of handgun use, we split our sample into those events involving handguns, and those not involving handguns (similar to the analysis with children). We then replicate our estimations of the market movement model. Results in Table 10 indicate that the negative market reaction disappears if the shooting does not involve a handgun. Results further indicate that if a handgun is involved there is a significant penalty for firearm manufacturers. Interestingly, results of the seemingly unrelated regression to examine the differences in coefficients across the subsamples suggest that only the results from the 2 day window are significantly different from each other (i.e. Columns 2 and 6). However, it is still insightful to note that absence of any significant effects in the sample where no handguns were used. The use of a handgun would indicate to the market that there is greater potential for regulatory action, since the event makes this particular aspect of handgun regulation salient. Alternatively, the use of other types of firearms is less likely to be affected by regulatory reactions. Thus, we see increasing support for the key mechanism being the threat of impending regulation, a key factor in the context of handguns.

**Table 10:** Effect of mass shootings on market returns, sample split based on whether or not a handgun is involved.

	(1)	(2)	(3)	(4)	(5)	(6)	(7)	(8)
Dependent Variable	r	r	r	r	r	r	r	r
Time Window	1 Day	2 Day	5 Day	10 Day	1 Day	2 Day	5 Day	10 Day
Sample	No Handgun				Handgun			
Treatment	-0.00208	0.000526	-0.00105	-0.00121	-0.00373	-0.00750**	-0.00357*	-0.00298*
	(0.00545)	(0.00373)	(0.00253)	(0.00158)	(0.00305)	(0.00319)	(0.00185)	(0.00159)
Market Return	1.443***	1.051***	1.009***	1.081***	0.940***	1.090***	1.066***	1.107***
	(0.273)	(0.224)	(0.141)	(0.0993)	(0.131)	(0.119)	(0.0801)	(0.0558)
Constant	0.00275	0.000398	0.000982	0.00218**	0.00335**	0.00454**	0.00204*	0.00313***
	(0.00351)	(0.00284)	(0.00156)	(0.00110)	(0.00162)	(0.00181)	(0.00107)	(0.00101)
N	174	290	638	1,218	378	630	1,386	2,646
R-squared	0.206	0.159	0.156	0.189	0.187	0.204	0.201	0.203

Dependent Variable: Percent Change in Firm Stock Price.

Bootstrapped standard errors in parentheses.

*** p<0.01

** p<0.05

* p<0.1.

### Location of the mass shooting

We next consider the influence of the location of the event on the market response. As discussed previously, legislation relating to the regulation of firearms is not only enacted at the federal level, but at the state and municipal level as well. Therefore, the level of current state and municipal regulation concerning firearms is likely reflected in the majority views of the voting population of the region, i.e., stronger local regulation in liberal regions in the US like New York, Massachusetts, and Washington DC but weaker regulation in conservative regions like Texas, Idaho, and Oklahoma. If state and local regulation is critical, then there are likely to be differences in the market’s response based on where the shooting occurs. Indeed, some emerging work attests to the fact that legislators in states that lean ideologically left vs. right tend to have a different response to mass shootings in terms of the gun-related bills that are introduced in those states (as a result of the increased salience that such events lend to firearms in general [15]). If such trends are observable over time, it would imply a differential response to mass shootings in terms of firearm stocks, contingent on the location of the event. We devise a test to check for differences in the market’s response based on the region’s ideological leanings to examine if market responses are likely associated with calls for regulation at the federal level, and not because of heterogeneous local regulation.

We use the Cook Partisan Voting Index (http://cookpolitical.com/) to account for the ideological leanings of the region, at the level of the state. Used extensively in prior research [74, 75], this variable provides a continuous indicator of the degree of partisanship (i.e. Democrat vs. Republican) in each state bounded between -40 (extreme liberalism) and +40 (extreme conservativism). 0 indicates a politically neutral location. We interact this variable with the treatment to determine if the reaction varies based on the partisan voting level of the location. Results, Table 11, indicate no significant moderating effect across location, thereby suggesting that regulation at the federal level (which legally supersedes state and municipal regulation and would therefore be applied unilaterally) is more likely to be instrumental here in terms of the market response. In addition, if the reductions in stock prices are associated with strategic divestment of firearm stocks by socially minded investment managers, such as those observed in the context of tobacco, gaming, and alcohol [20], the ideological bent of the location may be irrelevant. The market is location-agnostic and applies corrective action regardless, based on expectations of regulation.

**Table 11:** Effect of mass shootings on market returns moderated by cook partisan voting index.

	(1)	(2)	(3)	(4)
Dependent Variable	r	r	r	r
Time Window	1 Day	2 Day	5 Day	10 Day
Treatment	-0.00264	-0.00483**	-0.00262	-0.00233**
	(0.00292)	(0.00210)	(0.00165)	(0.000930)
Cook PVI	-1.82e-05	-0.000217	-5.66e-05	-4.61e-05
	(0.000114)	(0.000150)	(7.87e-05)	(5.96e-05)
Treatment * Cook PVI	9.14e-06	0.000103	0.000147	8.49e-05
	(0.000252)	(0.000179)	(0.000133)	(9.35e-05)
Market	1.047***	1.075***	1.051***	1.101***
	(0.128)	(0.110)	(0.0873)	(0.0443)
Constant	0.00324*	0.00296**	0.00164	0.00278***
	(0.00179)	(0.00128)	(0.00101)	(0.000808)
Observations	552	920	2,024	3,864
R-squared	0.188	0.191	0.187	0.199

Dependent Variable: Percent Change in Firm Stock Price.

Bootstrapped standard errors in parentheses.

*** p<0.01

** p<0.05

* p<0.1.

### Temporal effects

While our tests have thus far indicated support for the thesis that the impending threat of regulation may be the mechanism behind the short-term response of the market, our dataset includes 93 events over four years. The expectations of regulation are unlikely to be equally strong across all these events, notably when no significant regulatory actions are observed from earlier events. As a result, investors may “learn” over time that though there is likely to be a call for regulation after each event, the odds of follow-through are slim. Alternatively, it is possible that society adjusts to the “new normal” whereby mass shootings are not associated with any increased risk of regulation [76, 77]. Our dataset starts in 2009, closely following the election of President Barack Obama, when expectations of regulation were extremely high due to the Obama presidential campaign [33] and market responses to mass shootings may have been particularly strong. However, if the threat of regulation wanes after several years without any concrete actions on the part of the regulators, the market would ideally discount any such eventualities. We test for this in our dataset by dividing the sample into two groups representing early and later events, and estimating the market model within each chronological group.

Results, shown in Table 12, indicate that the negative responses are significant and negative only in the early period of the sample. Although results from the seemingly unrelated regression once again indicate a lack of significant differences in the coefficients across these two samples (as was the case with the handguns results), the absence of any significant difference from zero in the second 48 events is telling. Later mass shootings fail to generate any significant abnormal response from the market, suggesting that the market and investors had systematically discounted any threat of impending regulation in the latter half of the sample. The implications of these results are demoralizing in that they suggest mass shootings have indeed become the “new normal”, and that there no longer appears to be any expectation of regulatory intervention associated with them, although the effect on gun purchases remains strong [6]. An alternative view, of course, is that changes in government or legislature that may be associated with more or less potential for gun control could bring back the propensity of the market to respond abnormally. There is prior work arguing that demand for firearms is influenced by the increased possibility of regulation tackling firearms in the short-term, as shown by Bice and Hemley [78] in the context of the 1968 Gun Control Act and the Brady Act. However, this demand is contingent on perceptions of whether these policies or regulations are permanent or temporary. In our analysis, we see that making the possibility of gun regulation relatively less probably over time leads to significant attenuation of the market response, but these changes are reversible and need not always represent a permanent “normal.”

**Table 12:** Effect of mass shootings on market returns, sample split based on early versus late events.

	(1)	(2)	(3)	(4)	(5)	(6)	(7)	(8)
Dependent Variable	r	r	r	r	r	r	r	r
Time Window	1 Day	2 Day	5 Day	10 Day	1 Day	2 Day	5 Day	10 Day
Sample	First 48 Events in Sample				Second 48 Events in Sample			
Treatment	-0.00450	-0.00693***	-0.00464**	-0.00381***	-0.000854	-0.00293	-0.000738	-0.00104
	(0.00382)	(0.00213)	(0.00204)	(0.00131)	(0.00517)	(0.00381)	(0.00190)	(0.00163)
Market Return	1.009***	1.214***	1.303***	1.256***	1.049***	0.983***	0.924***	1.025***
	(0.134)	(0.0703)	(0.0800)	(0.0585)	(0.223)	(0.153)	(0.0867)	(0.0733)
Constant	0.00182	0.00248*	0.00222*	0.00278***	0.00471	0.00415*	0.00126	0.00290***
	(0.00187)	(0.00145)	(0.00116)	(0.000875)	(0.00290)	(0.00212)	(0.00134)	(0.00111)
N	276	460	1,012	1,932	276	460	1,012	1,932
R-squared	0.196	0.239	0.228	0.203	0.183	0.158	0.166	0.199

Dependent Variable: Percent Change in Firm Stock Price.

Bootstrapped standard errors in parentheses

*** p<0.01

** p<0.05

* p<0.1.

## Discussion and conclusion

In this work, we investigate how mass shootings in the US influence the stock price of publicly traded firearms manufacturers. Drawing upon research from stakeholder theory [51], and building on work showing that mass shootings appear to lead to an increase in the sales of firearms [6], we argue that an *a priori* relationship between these two events is unclear. On one hand, the increasing demand for guns, as a source of protection and a fear of impending regulation, should lead to a positive impact on the valuation of the firm. On the other, financial markets may respond negatively to such mass shootings because the long-term viability of the firms’ business models appears questionable and each event represents a renewed call for regulation. Results indicate that when mass shootings occur, there is a systematic negative reaction in the stock price of firearm manufacturers over a 2, 5, and 10-day window. Moreover, we observe that the context of the crime can moderate this penalty. While the number of victims and use of a handgun significantly increases the adverse reaction from the market, we see no effect on stock prices if the event involves children or if it happens in a democratic or republican state. Finally, we see that over time, this negative effect has faded, likely as a result of investors predicting (or learning) that no such action will materialize.

Taken in sum, results strongly suggest that when mass shootings occur, investors appear to be reducing their valuations of these firms. We contend that these results are reflective of the systematic violations of the social contract existing between firms and society in the US as a result of gun violence. Economically, our results indicate a penalty between 49.5 and 22.4 basis points, per day, when shootings occur, resulting in lost market capitalization of $5mm ($6.7mm) for Smith & Wesson (Ruger) over a 2-day window. Interestingly, these negative effects are not extended to other firms or industries that are closely associated with the two firearm manufacturers we study.

From a theoretical perspective, the context we study poses an interesting departure from much of the literature that addresses the economic costs of wrong-doing by organizations. On one hand, received research includes several studies that address the negative impact on a firm or on an industry when there is significant evidence of corporate wrong-doing or fraud [17, 79, 80]. Punitive action on the part of regulatory agencies or consumers in such cases is understandable and often takes the form of sanctions. Furthermore, the implications may even extend to relatively innocent but similar firms [19]. Alternatively, firms often experience negative attention due to industrial accidents, such as in the case of the BP Oil Spill in 2010 [81] or toys recall in 2007 [82]. In such cases, depending on the firm’s response to the crisis, there may even be a positive reputational effect accruing to the firm. For example, the decisions made by the executives at Johnson & Johnson to withdraw millions of bottles of contaminated Tylenol medication, at great cost, actually provided the firm considerable positive reputational capital, even though several patients died as a result of the contamination. In each of the above contexts, the focal firm had considerable agency and clearly bore responsibility for deliberate or accidental wrong-doing.

In the case of firearm manufacturers, especially in the US, there are significant differences in terms of corporate agency as well as responsibility. In many ways, the firearms industry is closer to the tobacco and alcohol industries in that they offer a product that is legal and regulated. However, the products marketed by tobacco and alcohol firms still require some level of individual agency on the part of the actual consumer. In contrast, with mass shootings, a disproportionate cost is borne by individuals who are neither customers nor directly related to the firearm industry. Moreover, there is rarely ever admittance of corporate wrong-doing nor are mass shootings considered to be industrial accidents. In spite of these events, firearms enjoy a level of prestige and acceptance that are rarely ever accorded to tobacco and alcohol firms, and operate in a context that is historically and economically unique to the US.

While a historical analysis of firearms in the US is beyond the scope of our analysis, the economic implications of how these products are used can and should be studied [2]. While much has been written about the health-related and criminological aspects of gun violence, there is relatively less work addressing how the markets interact with firearm manufacturers. Perhaps simply because firearm manufacturers have realized the social costs that may emerge from misuse of their products, most American firearm manufacturers are private and many are partly owned by private equity (e.g. the Cerberus Group [83]). If the market is truly a channel through which social norms are reflected, and is also a medium by which social contracts between firms and society are governed (i.e. firms are implicitly sanctioned or rewarded), most firearm firms simply exist beyond these channels. They operate in a region where on the one hand, breaches of the social contract are irrelevant to the firms *per se*, and, on the other, society has limited means by which it can sanction these firms. This is reflected in prior work that has addressed first, the limited attempts made by firearm firms to apply the right safeguards to their products so as to reduce misuse [54], and second, the abilities of the firearms firms to lobby and influence government and policy makers [1]. Our work is the first to provide a simple but compelling cost estimate of the penalty extended to firearm firms, even in a limited way, by markets for bearing “moral responsibility” for shootings [55].

In terms of practical implications, our work here helps inform policy makers about some of the costs associated with mass shootings, and therefore gun violence, in the US. Much of the gun violence in the US occurs outside such mass shootings and therefore, in real terms the economic costs we identify are relatively small. However, it is worth considering what the total cost of gun violence is likely to be if all such costs could be reflected through the market. With just two firms, we observe losses in market capitalization of over $70M. If more firearm firms were public or the true costs of gun violence in other contexts could be quantified, there would likely be a cleaner case made for reasonable gun regulation and control that would satisfy all parties. That said, our analysis also reflects an uncomfortable reality–that the market adjusts to non-action on the part of regulatory agencies by systematically under-responding to such events. The market’s response is rational, if distressing from a societal and ethical perspective, since the risk of regulation has been observed to be systematically reducing in the US during the period of our analysis [33].

A further implication of our work, albeit in a different context, would be to inform political entities of the value of gun control legislation that may limit both the human and economic costs of mass shootings. Recent work has attempted to causally link mass shooting events to the introduction of bills in legislative bodies within the United States, seeking to address restrictions on firearms [15]. In this research, the authors argue that the salience of mass shootings does tend to increase the number of bills that are created, relative to other firearm-related homicides. This is consistent with our findings that shootings with a greater number of victims elicits a stronger response. However, whether these bills are enacted or not is likely driven by the political climate in that state; with Republican-controlled states appearing to loosen restrictions following mass shootings and Democrat-controlled states showing little to no effects. Although it may be possible to reverse this argument and control for the presence of proposals targeting gun control in our model, under the belief that the stock market’s response may be stronger if gun control regulations were actively being debated in legislative bodies, this is infeasible since we are interested in short-term market responses to the mass shootings, and regulations tend to change slowly. Further, these would be correlated with the state-level controls we apply in our regression (such as Cook’s DVI). Nevertheless, we believe there are several opportunities for additional research addressing the relationship between mass shootings and legislative activities; we leave these for future work.

Our work here has certain limitations. First, we are limited by the fact that only two publicly traded firearms firms are available for analysis in the US. Given this small set, it may be reasonable to consider our work here as a case study rather than a generalizable large-sample event study. However, as we use public data sources for our analysis, all of our models can be independently verified. Second, given the randomness of mass shootings and the exogeneity it provides, we estimate relatively simple models of stock movements after the event. While more complicated econometric models may be devised, we believe that the basic results will not change, given the sharply diverging coefficients observed for the S&P 500 index and the firearms stocks. Finally, while we theorize about social contract breaches and the risk of regulation in our work, we cannot rule out alternative explanations that may drive stock prices. Perfect identification of the mechanism is difficult, but the set of results we observe across multiple specifications provides some evidence of mechanisms. Future work is needed to fully establish whether regulation is indeed the dominant force.

In summary, in this paper we investigate how mass shootings in the US influence the stock price of publicly traded firearms manufacturers. Results strongly suggest that when mass shootings occur, investors appear to be reducing their valuations of these firms and it appears that this effect is driven by the threat of impending regulation. Economically, results indicate a penalty between 49.5 and 22.4 basis points, per day, when shootings occur, resulting in lost market capitalization of $11.3mm ($15.05mm) for Smith & Wesson (Ruger) over a 10-day window, particularly when handguns are used. Results also show that these effects appear to be increasingly attenuated in the later period of our dataset, indicating that investors do not expect any regulatory action with respect to gun control in the US in the later years of our analysis, implying possible acceptance of mass shootings as the “new normal”.

## Supporting information

S1 File Statistical appendix. (DOCX)
