# Supplementary material for: Traders, guns, and money: The effects of mass shootings on stock prices of firearm manufacturers in the U.S
Source: PLoS One. 2017 May 18;12(5):e0177720. doi: 10.1371/journal.pone.0177720 (PMC5436715; doi:10.1371/journal.pone.0177720)
Supplement: S1 File — (DOCX) [file pone.0177720.s001.docx]

Traders, Guns, and Money: The Effects of Mass Shootings on Stock Prices of Firearm Manufacturers in the U.S.

Anandasivam Gopal^1*¶^, Brad N. Greenwood^2¶^

^1^ Robert H Smith School of Business, University of Maryland – College Park, College Park, MD, USA

^2^ Fox School of Business, Temple University, Philadelphia, PA, USA

^*^ Corresponding Author

Email: agopal@rhsmith.umd.edu

^¶^ These authors contributed equally to this work.

# Statistical Appendix

In this Statistical Appendix, we provide results associated with the multiple robustness tests that were conducted for the baseline regressions reported in the main paper. The focus of these robustness tests are for the main effect of mass shootings on short-term firearm stock prices (those shown in Tables 2a-2c).

# Including Day 0 in the Event Study

Recall that in the results reported in the paper (Tables 3-5), we specifically exclude the date of the event to ensure that partial information dissemination does not influence the results. However, it is possible that the stock price increases rapidly immediately after the event occurs (i.e. on the 0^th^ day), and the negative downward trend we observe on day 1 is actually a market correction. Therefore, we conduct analysis where we include the 0^th^ day (i.e. the day of the shooting) to account for the possibility of market overcorrection on the day after the shooting. Results are shown in Table A1 and are fully consistent with those discussed earlier, showing a significant negative reaction from the market following the shooting along with a marginally significant effect on Day 1 (p<0.1). All subsequent analyses reported in the paper addressing the moderating factors were replicated including the 0^th^ day, and are fully consistent.

**Table A1.** Effect of Mass Shootings on Firearm Manufacturer Market Returns including Day 0 as Treated

|  | (1) | (2) | (3) | (4) | (5) |
| --- | --- | --- | --- | --- | --- |
| Dependent Variable | r | r | r | r | r |
| Time Window | 0 Day | 1 Day | 2 Day | 5 Day | 10 Day |
| Treatment | -0.00522 | -0.00524* | -0.00544*** | -0.00271* | -0.00263*** |
|  | (0.00319) | (0.00293) | (0.00174) | (0.00161) | (0.000958) |
| Market Return | 1.062*** | 1.052*** | 1.070*** | 1.050*** | 1.100*** |
|  | (0.177) | (0.110) | (0.0949) | (0.0670) | (0.0541) |
| Constant | 0.00588** | 0.00587*** | 0.00451*** | 0.00192* | 0.00306*** |
|  | (0.00269) | (0.00226) | (0.00145) | (0.00111) | (0.000801) |
| N | 368 | 552 | 920 | 2,024 | 3,864 |
| R-squared | 0.211 | 0.191 | 0.189 | 0.187 | 0.199 |
| Bootstrapped standard errors in parentheses | | | | | |
| *** p<0.01, ** p<0.05, * p<0.1 | | | | | |

# Constant 20-day Training Period for the Market Movement Model

In our baseline estimations shown in Tables 2a-2c, we use the same window of days pre-event to “train” the model (i.e. estimate the correlation between the firearm stock and the S&P 500 market index / DGTW index) as the number of days post-event to estimate the treatment effect. In other words, the 5-day model uses 5 days pre-event plus 5-days post-event plus the day of the event to estimate the model (totaling eleven days). This also leads to varying sample sizes used in each analysis reported for each time window. It is possible that using a balanced training set (i.e. the number of pre-treatment days is equal to the number of post treatment days), may lead to biased estimates (especially in the shorter time-periods because the shorter term training periods are not long enough to capture the true correlation with the market). We therefore replicate our estimations using a constant 20-day training period across all analyses. Results are in Table A2 and yield consistent results. Note that all the regressions reported elsewhere in the paper are replicated with the constant 20-day training period and results remain consistent.

**Table A2.** Effect of Mass Shootings on Firearm Manufacturer Market Returns using Consistent 20 Day Training Period

Dependent Variable: Percent Change in Firm Stock Price

|  | (1) | (2) | (3) | (4) |
| --- | --- | --- | --- | --- |
| Dependent Variable | r | r | r | r |
| Time Window | 1 Day | 2 Day | 5 Day | 10 Day |
| Treatment | -0.00270 | -0.00499*** | -0.00441*** | -0.00289*** |
|  | (0.00277) | (0.00172) | (0.00113) | (0.000732) |
| Market Return | 0.980*** | 0.987*** | 1.004*** | 1.023*** |
|  | (0.0593) | (0.0574) | (0.0563) | (0.0524) |
| Constant | 0.00343*** | 0.00342*** | 0.00341*** | 0.00340*** |
|  | (0.000543) | (0.000606) | (0.000532) | (0.000580) |
| N | 3,818 | 4,002 | 4,554 | 5,474 |
| R-squared | 0.153 | 0.155 | 0.162 | 0.170 |
| Bootstrapped standard errors in parentheses | | | | |
| *** p<0.01, ** p<0.05, * p<0.1 | | | | |

# Placebo Effects – The Impact of Celebrity Deaths

It is well established that firearm stocks are relatively volatile. Therefore, it is possible that the short-term price movements we observe are not actually related to mass shootings, but are based on structural volatility. In order to further examine these, we conduct a placebo analysis, wherein we look for significant price movement associated with events that are clearly unrelated. We use celebrity deaths that occurred in the United States during the time period of our analysis, and examine how firearm firm stock prices may be affected. We use notable 46 celebrity deaths gathered from the Hollywood Reporter, i.e. we replace the mass shooting dates with the deaths of these celebrities and re-estimate the model. The results, shown in Table A3, show a positive significant effect on the first day and no effect afterwards. Note that our original regressions showed a negative coefficient across the first five days of the treatment period (post-shooting). The placebo analysis shows that the obtained results are not incidental but capture the effects of the mass shootings.

**Table A3.** Replication using 46 Celebrity Deaths from the Hollywood Reporter

Dependent Variable: Percent Change in Firm Stock Price

|  | (1) | (2) | (3) | (4) |
| --- | --- | --- | --- | --- |
| Dependent Variable | r | r | r | r |
| Time Window | 1 Day | 2 Day | 5 Day | 10 Day |
| Treatment | 0.0107*** | 0.00597 | 0.00347 | 0.00318 |
|  | (0.00413) | (0.00382) | (0.00251) | (0.00199) |
| Market Return | 1.216*** | 1.259*** | 1.325*** | 1.216*** |
|  | (0.125) | (0.135) | (0.0910) | (0.0626) |
| Constant | 0.000361 | 6.18e-05 | 0.00138 | -0.000115 |
|  | (0.00245) | (0.00169) | (0.00180) | (0.00126) |
| N | 230 | 376 | 816 | 1,540 |
| R-squared | 0.233 | 0.218 | 0.213 | 0.195 |
| Bootstrapped standard errors in parentheses | | | | |
| *** p<0.01, ** p<0.05, * p<0.1 | | | | |

# Individual Results for RGR and SWHC

Our sample consists of two firearm manufacturers that are publicly traded in the US. While the results reported in the paper do not separate the firms, we provide individual firm-level analysis here as a robustness test. As shown in Table A4, the firm-level results are largely similar in terms of coefficients and significance. The betas do differ but the overall patterns of responses to mass shootings are similar.

**Table A4**. Individual Firm-level Analysis

Dependent Variable: Percent Change in Firm Stock Price

|  | (1) | (2) | (3) | (4) | (5) | (6) | (7) | (8) |
| --- | --- | --- | --- | --- | --- | --- | --- | --- |
| Dependent Variable | r | r | r | r | r | r | r | r |
| Time Window | 1 Day | 2 Day | 5 Day | 10 Day | 1 Day | 2 Day | 5 Day | 10 Day |
| Sample | RGR | | | | SWHC | | | |
| Treatment | -0.00298 | -0.00486* | -0.00261* | -0.00218* | -0.00233 | -0.00505* | -0.00297 | -0.00269** |
|  | (0.00340) | (0.00260) | (0.00142) | (0.00131) | (0.00466) | (0.00321) | (0.00238) | (0.00134) |
| Market Return | 0.722*** | 0.790*** | 0.835*** | 0.902*** | 1.371*** | 1.361*** | 1.267*** | 1.299*** |
|  | (0.150) | (0.112) | (0.0921) | (0.0516) | (0.200) | (0.160) | (0.106) | (0.0765) |
| Constant | 0.00190 | 0.00112 | 0.00110 | 0.00247** | 0.00463* | 0.00532*** | 0.00231* | 0.00320*** |
|  | (0.00195) | (0.00163) | (0.00108) | (0.00103) | (0.00258) | (0.00194) | (0.00136) | (0.00112) |
| N | 276 | 460 | 1,012 | 1,932 | 276 | 460 | 1,012 | 1,932 |
| R-squared | 0.138 | 0.149 | 0.169 | 0.172 | 0.240 | 0.231 | 0.208 | 0.227 |
| Bootstrapped standard errors in parentheses | | | | | | | | |
| *** p<0.01, ** p<0.05, * p<0.1 | | | | | | | | |

# Using Net Return Over S&P 500 Market Index as the Dependent Variable

It is possible to conduct the analysis by considering, as the dependent variable, the extent to which the firearm stock prices move above and beyond the relative changes in the S&P 500 Market Index. In such an analysis, the dependent variable is the actual changes in the stock price, as opposed to the percent daily change in the stock price. We conduct such an analysis using the S&P 500 index and the results, shown in Table A5, show patterns that are consistent with those seen before. Note that the beta here is in stock price terms, rather than in terms of % change.

**Table A5**. Net Returns of Firearm Stocks over the S&P 500 Market Index

Dependent Variable: Change in Actual Stock Price

|  | (1) | (2) | (3) | (4) |
| --- | --- | --- | --- | --- |
| Dependent Variable | Net Returns | Net Returns | Net Returns | Net Returns |
| Time Window | 1 Day | 2 Day | 5 Day | 10 Day |
| Treatment | -0.0613 | -0.0864** | -0.0517* | -0.0462** |
|  | (0.0544) | (0.0416) | (0.0339) | (0.0196) |
| Market Return | 10.68*** | 12.03*** | 12.41*** | 12.61*** |
|  | (1.538) | (1.426) | (0.954) | (0.642) |
| Constant | 0.0140 | 0.00852 | 0.00997 | 0.0303** |
|  | (0.0309) | (0.0254) | (0.0203) | (0.0135) |
| N | 552 | 920 | 2,024 | 3,864 |
| R-squared | 0.060 | 0.068 | 0.074 | 0.075 |
| Bootstrapped standard errors in parentheses | | | | |
| *** p<0.01, ** p<0.05, * p<0.1 | | | | |

An alternative approach would be to use *r* as the dependent variable but consider the change in stock price, relative to the changes in the prices of the S&P 500 Market Index. Thus, r is defined as the % change in firm price minus the % change in the S&P 500. We present the results from analysis in Table A6, showing consistent and robust results. We thank an anonymous reviewer for suggesting the tests in Table A5 and A6.

**Table A6**. Effect of Mass Shootings on Stock Price Incorporating Market Movement Change into Dependent Variable

Dependent Variable: % Δ in Firm Price - % Δ in S&P 500

|  | (1) | (2) | (3) | (4) |
| --- | --- | --- | --- | --- |
| Dependent Variable | Δ Between r and Market Return | Δ Between r and Market Return | Δ Between r and Market Return | Δ Between r and Market Return |
| Time Window | 1 Day | 2 Day | 5 Day | 10 Day |
| Treatment | -0.00255 | -0.00488* | -0.00274* | -0.00235*** |
|  | (0.00268) | (0.00255) | (0.0017) | (0.000795) |
| Constant | 0.00324* | 0.00329** | 0.00175 | 0.00290*** |
|  | (0.00180) | (0.00151) | (0.00111) | (0.000723) |
| N | 552 | 920 | 2,024 | 3,864 |
| R-squared | 0.001 | 0.005 | 0.002 | 0.001 |
| Bootstrapped standard errors in parentheses | | | | |
| *** p<0.01, ** p<0.05, * p<0.1 | | | | |

# Description of the TNIC Industry Classification

TNIC industry classifications are based on the proposition that firms in the same industry use many of the same words to describe their products in the business description section of firm 10-Ks. Using 10-K text offers many advantages including improved signal strength, the ability to measure competition dynamically even when the product market changes rapidly, and the ability to use a general intransitive network structure. These features have proven useful in other settings including: research on mergers, asset prices, payout policy, and firm organizational form. Moreover, they are particularly well suited for use in IT industry, which is known for rapid change.

In order to construct TNIC industries, Hoberg and Phillips (2010a, 2010b) web crawl all available 10-Ks from the SEC’s Edgar website and link each 10-K to the Compustat database in each year using the central index key (CIK) as the unique firm identifier. A link table from CIK to Compustat gvkey is provided by the Wharton Research Data Service (WRDS) through the SEC Analytics package. Once business description sections are parsed from each 10-K, common words (those appearing in more than 25% of all 10-Ks in a given year) are discarded. Additionally, any word that does not appear as a noun or proper noun is also discarded. The typical firm uses 200 unique words, and any firm using fewer than 20 is omitted.

Words are then mapped to numerical vectors and firm pairwise cosine similarity scores are computed for every pair of firms in each year (cosine similarities are a popular tool in computational linguistics, see Sebastini [1] for example). In particular, each firm *i*'s vocabulary can be represented by a vector *P_i_*, which has a length equal to the number of unique words, with each element being populated by the number one if firm *i* uses the given word, and zero if it does not. These vectors are then normalized to have unit length (which is done by dividing all elements of the vector by the square root of the vector’s dot product with itself). The normalized vectors are denoted as *V_i_*. Because all firms are thus represented by vectors of length one in the same space, it follows that TNIC industries imply that firms have unique locations on a high dimensional unit sphere, and that they move along the surface of the sphere as their products are revised from year to year.

To identify peers in the TNIC network, the cosine similarity between pairs of firms *i* and *j* are calculated as follows:

$${Cosine Similarity}_{i,j}=(V_{i}\cdot V_{j})$$

The full TNIC network is thus fully described by an *NxN* square matrix that is populated with cosine similarities between all firms in each year, where *N* is the number of firms. Due to the properties of cosine similarities, all entries are real numbers in the interval [0,1]. Because firms update their 10-Ks annually, the network (the entire square matrix) is time-varying. In the current article, we consider the TNIC-3 network, which is calibrated to be as granular as is the three-digit SIC code network (SIC-3). This is done by deeming the firm pairs having cosine similarities that are among the highest 2.05% of similarities as being rivals, as SIC-3 implies the same fraction of all firm pairs are rivals.

TNIC industries have many important features. First, tests in Hoberg and Phillips [2, 3] illustrate that they are more informative than other frequently used industry classifications such as SIC or NAICS code. Second, the entire classification is dynamically updated every year because firms file updated 10-Ks every year as required by regulation S-K. Third, TNIC industries are part of a generalized intransitive network, providing much research flexibility to consider customized sets of rivals for each firm. In contrast, classifications such as SIC codes are less powerful, rarely updated, and are constrained to be transitive.

In a robustness test, we replicate the estimation using SIC-3 classification rather than the TNIC analysis reported in the paper. The results are provided in Table A7. We see the treatment effect on the first two days immediately after the event being positive, and highly correlated with the market index. The industry category includes several firms that are only marginally related to the industry we study, such as those in ordnance, artillery, and missile warheads (SIC 348). Clearly, these firms are unrelated to the small arms industry that we study. Additionally, the estimated ‘beta’ with the S&P market index for the firearms firms in our analysis is roughly 1.05 across all the analyses. In the regression where we use the TNIC firms, the beta is roughly of the same magnitude (1.049-1.18). However, in the analysis using SIC-3 firms, the beta falls to roughly half (0.44 – 0.57), again indicating that these firms are not very similar to the firearms firms we study. The results here provide support for the use of the TNIC rather than the SIC-3.

**Table A7.** Effect of Mass Shootings on SIC Market Returns

Dependent Variable: Percent Change in Firm Stock Price

|  | (1) | (2) | (3) | (4) |
| --- | --- | --- | --- | --- |
| Dependent Variable | r | r | r | r |
| Time Window | 1 Day | 2 Day | 5 Day | 10 Day |
| Treatment | 0.0152** | 0.0114** | 0.00350 | 0.000937 |
|  | (0.00634) | (0.00449) | (0.00296) | (0.00257) |
| Market Return | 0.445*** | 0.481*** | 0.507*** | 0.579*** |
|  | (0.162) | (0.153) | (0.0909) | (0.0660) |
| Constant | 0.00117 | -0.000487 | 0.00336 | 0.00497*** |
|  | (0.00402) | (0.00294) | (0.00204) | (0.00143) |
| N | 1,618 | 2,692 | 5,922 | 11,309 |
| R-squared | 0.006 | 0.006 | 0.004 | 0.004 |
| Bootstrapped standard errors in parentheses | | | | |
| *** p<0.01, ** p<0.05, * p<0.1 | | | | |

# References

1. Sebastiani F. Machine learning in automated text categorization. ACM Computing Surveys. 2002;34(1):1-47.

2. Hoberg G, Phillips G. Real and financial industry booms and busts. The Journal of Finance. 2010;65(1):45-86.

3. Hoberg G, Phillips GM. Text-Based Network Industries and Endogenous Product Di fferentiation. National Bureau of Economic Research, 2010.
